# Kinase Inhibitor Therapy in CML: It's What's Inside That Counts

**DOI:** 10.18632/oncotarget.1271

**Published:** 2013-08-09

**Authors:** Christopher A. Eide, Brian J. Druker, Thomas O'Hare

**Affiliations:** Howard Hughes Medical Institute; Division of Hematology and Medical Oncology, Oregon Health & Science University, Knight Cancer Institute, Portland, OR; Howard Hughes Medical Institute; Division of Hematology and Medical Oncology, Oregon Health & Science University, Knight Cancer Institute, Portland, OR; Huntsman Cancer Institute; Division of Hematology and Hematologic Malignancies, The University of Utah, Salt Lake City, UT

The paradigm-shifting clinical success of imatinib in the treatment of chronic myeloid leukemia (CML) established continuous inhibition of the BCR-ABL1 oncoprotein as a design principle for ABL1 tyrosine kinase inhibitors. However, once-daily dosing with the second-generation ABL1 inhibitor dasatinib, which features a short serum half-life of only 3-5 h, was found to be clinically effective despite apparent transient BCR-ABL1 inhibition [1]. This suggests involvement of previously unrecognized factors among the mechanistic requirements for ABL1 tyrosine kinase inhibitor-induced CML cell death.

Previous work by our group and others demonstrated that apoptosis commitment after potent, transient target inhibition can be recapitulated using in vitro inhibitor washout protocols [2-4]. One hypothesis that has been proposed to explain these findings – oncogenic shock – holds as its central tenet that temporary, potent disruption of BCR-ABL1-mediated prosurvival and proapoptotic signaling sets up an irreversible kinetic imbalance in favor of apoptosis [5]. This situation can be likened to a tightrope walker who is swept from his perch by a sudden crosswind, sealing his plight. Such a model implies that programmed cell death is guaranteed following brief shut-off of oncogenic kinase activity despite reactivation of kinase signaling and removal of inhibitor from the system.

In the June 1, 2013 issue of *Cancer Research* [6], we provide a comprehensive mechanistic exploration of the effects of transient inhibitor exposure. We treated CML cells transiently with a panel of five clinically-relevant ABL1 tyrosine kinase inhibitors – imatinib, nilotinib, dasatinib, ponatinib (AP24534), DCC-2036 – and investigated pathways critical to drug efficacy and intracellular residence time, focusing on clinically-relevant concentrations of each drug. Dasatinib, nilotinib, and ponatinib were capable of triggering apoptosis following transient exposure; neither imatinib nor DCC-2036 induced significant apoptosis following washout of concentrations up to 5 μM. In contrast to potent, transient inhibition of BCR-ABL1 being the only requirement for commitment of CML cells to apoptosis, we found that apoptosis could be reversed under conditions involving extensive additional inhibitor washout. Multi-parameter intracellular FACS and immunoblot analysis revealed that commitment to apoptosis following washout tracked with incomplete restoration of BCR-ABL1 signaling relative to pretreatment levels, particularly with respect to phosphorylation of STAT5. In all cases for which apoptosis commitment was observed, we identified by liquid chromatography-tandem mass spectrometry (LC-MS/MS)-based assay a small, functionally important pool of intracellular inhibitor retained after washout of drug. Conditions under which apoptosis commitment could be mitigated or completely rescued by more extensive drug washout were associated with decreased intracellular levels of inhibitor post-washout and full restoration of BCR-ABL1 signaling. ABL1 kinase:inhibitor dissociation studies revealed differences in binding off-rates among the tested inhibitors, which coincided with protracted partial inhibition of BCR-ABL1 signaling and the fraction of intracellular drug removed with a given washout protocol. Most notably, ponatinib demonstrated extremely tight binding to ABL1 kinase reminiscent of irreversible inhibitors. Low amounts of residual ponatinib in CML cells following extensive washout were capable of inducing substantial apoptosis and sustaining partial inhibition of BCR-ABL1 signaling.

**Figure 1:**
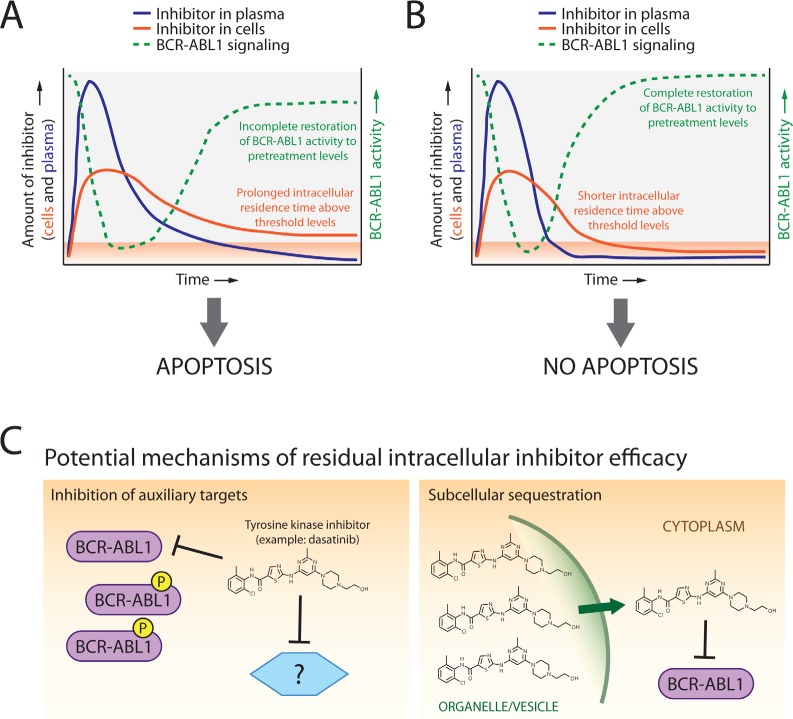
Model of dynamics, distribution, and mechanisms of ABL1 tyrosine kinase inhibitor-mediated apoptosis commitment in CML cells following transient drug exposure A. For tyrosine kinase inhibitors which feature short plasma half-lives (blue lines), screening for and explanation of potential efficacy may require determination of levels of drug retained in cells (orange lines). Inhibitors capable of inducing subsequent apoptotic cell death demonstrate protracted intracellular retention above threshold levels (orange shaded area) along with incomplete restoration of BCR-ABL1 kinase signaling activity (green lines). B. In contrast, inhibitors that are rapidly cleared from both the plasma and cells show complete restoration of BCR-ABL1 activity relative to pretreatment levels and do not commit cells to apoptosis. C. Potential mechanisms by which threshold-exceeding levels of retained ABL1 tyrosine kinase inhibitors trigger apoptosis despite only partial inhibition of BCR-ABL1 activity may include inhibition of auxiliary targets that reinforce pro-apoptotic signaling (left panel) and/or significant accumulation of inhibitor within select compartment(s) of the cell from which drug is gradually released (right panel).

Our findings reveal that even slightly attenuated restoration of BCR-ABL1 signaling correlates with apoptosis commitment and that intracellular retention of ABL1 tyrosine kinase inhibitors above a quantifiable threshold is important in mediating this effect (Figure 1A,B). Other groups have reported corroborating results for imatinib and dasatinib [7, 8]. However, the complete details underlying how the residual intracellular inhibitor pool exerts its apoptotic effects despite only partial to minimal sustained inhibition of BCR-ABL1 kinase remains unknown. The situation is not so black and white as to indicate that oncogenic shock is a fallacy and that cryptic intracellular drug retention explains all. Rather, there may be a nuanced collaboration between these explanations. One possibility is that auxiliary targets may be also inhibited by low levels of retained inhibitor (Figure 1C, left panel). In high-throughput qPCR assays using a panel of >600 apoptosis-related genes, we observe that CML cells under apoptosis-triggering treatment conditions feature highly similar expression profiles irrespective of whether resulting from acute or continuous drug exposure. This would suggest that if an auxiliary target is important and inhibited, it does not activate unique additional apoptotic machinery under acute drug exposure conditions (unpublished data). Since the LC-MS/MS method measured the total amount of inhibitor retained within the entire volume of the cell, it is also possible and perhaps likely that the distribution of residual inhibitor within the cell is non-uniform [9], leading to compartmental sequestration and gradual leaching out of inhibitor over time (Figure 1C, right panel).

Addressing these and similar questions will further inform design requirements for ABL1 tyrosine kinase inhibitors in CML. It appears clear that metabolic half-life must continue to be taken into account when designing kinase inhibitors, dampening the promise of the oncogenic shock concept. By extension, monitoring intracellular drug levels by LC-MS/MS may be informative, especially for short serum half-life kinase inhibitors such as dasatinib. The cumulative effort to distill the essential properties of effective small-molecule kinase inhibitors will accelerate identification and development of targeted therapies with improved efficacy and tolerability for CML and other malignancies.
